# Comparison of Fluoride Release in Conventional Glass- Ionomer Cements with a New Mechanical Mixing Cement

**DOI:** 10.3290/j.ohpd.a44034

**Published:** 2020-07-04

**Authors:** Adriana A Morales-Valenzuela, Rogelio J Scougall-Vilchis, Edith Lara-Carrillo, Rene Garcia-Contreras, Elias N Salmeron-Valdes, Lizzeth Aguillón-Sol

**Affiliations:** a Part-time Professor, Autonomous University State of Mexico, Mexico. Wrote the manuscript, performed the experiments and statistical evaluation, revision of the manuscript.; b Full-time Professor and Chairman, Dental Research Center ‘Dr. Keisaburo Miyata’, School of Dentistry, Autonomous University State of Mexico, Mexico. Study concept and idea, proofreading and revision of the manuscript.; c Full-time Professor and Dean, Dental Research Center ‘Dr. Keisaburo Miyata’, School of Dentistry, Autonomous University State of Mexico, Mexico. Contributed substantially to discussion, revision of the manuscript.; d Full-time Professor, Universidad Nacional Autónoma de México, Mexico. Experimental design, revision of the manuscript.; e Part-time Professor, Universidad Nacional Autónoma de México, Mexico. Experimental design, revision of the manuscript.; f Part-time Professor, Dental Research Center ‘Dr. Keisaburo Miyata’, School of Dentistry, Autonomous University State of Mexico, Mexico. Assisted with experiments, revision of the manuscript.

**Keywords:** anticariogenic agents, fluoride, glass-ionomer cement

## Abstract

**Purpose:**

The aim of this paper was to compare three conventional hand mixing glass-ionomer cements (GICs) and a new mechanical mixing glass-ionomer cement.

**Materials and Methods:**

Samples were measured on days 1, 2, 6, 10, 31, 90 and 180. After 32 and 181 days of monitoring, the samples were recharged by using 1 ml of 2% sodium fluoride gel.

**Results:**

The fluoride release started in high concentration during the first day for all GICs, with a value for GIII of 32.6 ppm. From the 2nd day, a slow, steady decline, with the exception of GII, which showed a marked decline to a value of 3.2 ppm. Analysis of variance (ANOVA) test showed statistically significant differences between the amounts of fluoride of the four materials in the first 24 h. Student t test was used to compare the fluoride release between the first and second recharge in each one of the study groups. Statistically significant differences were found when we compared the fluoride release in groups I (t = –16.95, p = 0.000) and IV (t = –2.644, p = 0.26).

**Conclusions:**

A mechanical mixing was the material with the more constant fluoride release and after recharge showed the highest fluoride release which make it an important benefit for clinicians.

The mechanism of glass-ionomer cements (GICs) is an acid-base reaction between ion-leachable fluoroaluminosilicate glasses and polyalkenoic acids.^11,18,23,25^

One mechanism is a short-term reaction, which involves rapid dissolution from outer surface into solution, whereas the second is more gradual and results in sustained diffusion of ions through the bulk cement.^19,26^

The fluoride is well documented as an anticariogenic agent.^1,15,26^ The anticariogenic effect of fluoride-releasing materials depends on the amount and sustainability of fluoride release. The fluoride release from a restorative material is determined by the matrix of the restorative material, the mechanism by which it sets, and the amount of fluoride-containing fillers.^16,17^ A variety of mechanisms is involved in the anticariogenic effects of fluoride, including the reduction of demineralisation, the enhancement of remineralisation, the interface of pellicle and plaque formation, and the inhibition of microbial growth and metabolism. Fluoride released from dental restorative materials is assumed to affect caries formation through all these mechanisms and may therefore reduce or prevent demineralisation and promote remineralisation of dental hard tissues.^9,10,^^26^ Previous studies have demonstrated that variables intrinsically related to the chemical formulation, as well as to the physical presentation of the GICs, affect the fluoride release qualitatively and quantitatively. These variables, such as the composition of the aluminosilicate glass and the polyalkenoic acid, the particle size of the glass powder, the relative proportion of the constituents (glass/polyacid/tartaric acid/water) in the cement mix and the type of mixing, are mainly determined by the manufacturer.^6,7^

Some studies indicate that hand mixing and mechanical mixing in capsules can result in a different fluoride release profile, suggesting that the mixing process could play an important role.^7^ Recently, it has been reported that fluoride-releasing materials can take up fluoride ions from the oral environment as a means of replacing fluoride which has been lost. The recharge of fluoride may contribute to the ability of these materials to provide a long-term inhibitory effect on enamel demineralisation, because the recharged fluoride is released again and presumably contributes to continuous prevention of enamel demineralisation.^2,13,21,22^ Therefore, the objective of this study was to compare three conventional hand mixing GICs with a new mechanical mixing glass-ionomer cement.

## MATERIALS AND METHODS

### Specimen Preparation

For conventional hand mixing, GICs were used in this study: a GII-Fuji IX (GC, Kyoto, Japan); GIII-Ionofil Molar (Vocco, Cuxhaven, Germany); GIV-Ketac Molar (3M Oral Care, St Paul, MN, USA). For the mechanical mixing group, the glass-ionomer cement was GI-Fuji IX GP EXTRA (GC).

The materials were handled according to the manufacturers’ instructions, and 40 samples were prepared. The samples consisted of 10 blocks of each GICs with 5 mm width and 1 mm thickness; the samples were placed in cavities with similar measures in a Teflon matrix.^8,12^

The polymerised samples were removed from the matrix and later stored in plastic bottles with 5 ml of deionised water. The samples were conserved at 37°C for 60 days and measured on days 1, 2, 6, 10, 31, 90 and 180, which is similar to the time intervals used in previous studies.^8,12^

### Instrumentation and Reagent Solutions

To determine the amount of fluoride in GICs, it was necessary to use an ion-selective electrode for sodium fluoride (model 1011; Hanna Instruments, Ann Arbor, MI, USA) and a potentiometer (model 3222; Hanna Instruments). The total ionic strength adjustment buffer (TISAB) solution was used to keep the pH stable and to prevent the fluoride ion from producing complexes with different cations.^21^

### Potentiometer Calibration

The fluoride solutions used in this study were prepared in concentrations of 1, 2, 10, 100, and 1000 ppm. TISAB was used to obtain a calibration slope with fluoride solutions; equal volumes of fluoride solution and TISAB (25 ml of each) were placed and mixed in a 100-ml plastic glass; the device was calibrated until the readings were reached.^21^

### Fluoride Determination

At the end of each period, the blocks were removed from their respective recipients, and each sample was washed with 1 ml of deionised water in the bottle which was the original container. Five millilitres (5 ml) of solution was used to store the sample, and 1 ml was used to wash the sample, giving a total of 6 ml that was mixed with 6 ml of TISAB, because this solution works in a proportion of 1:1.

The sample was placed in a new 5 ml plastic bottle with deionised water.

The readings were performed under magnetic stirring for 3 min with the electrode immersed in the solution where the sample had been previously. The values of the readings were expressed in parts per million.^2,21^

After 32 and 181 days of monitoring, the samples were recharged using 1 ml of 2% sodium fluoride gel (Ionite Borgatta, Mexico). The samples were immersed in this gel for 4 min and subsequently cleaned with a sterile gauze. The fluoride released in the samples after recharge was determined daily for 5 days.^12,21^

The data were analysed with analysis of variance (ANOVA), and Student’s t test was used for related samples using the 21st version of the statistical program SPSS Statistics (IBM, Nashville, Tennessee).

The aim of this paper was to compare three conventional hand mixing GICs and a new mechanical mixing glass-ionomer cement. And the work hypothesis is ‘The fluoride release of the glass-ionomer cement reinforced with NPs of TiO_2_ is greater than that released by the conventional glass-ionomer cement’.

## RESULTS

The pattern of fluoride released according to the time intervals is represented in Table 1 and started with high concentration for the first day for all GICs, with a value for GIII of 32.6 ppm, which makes this material the one with the highest fluoride concentration, and for GIV, it presented fluoride releases of 17.4 ppm, which makes it the GIC with the lowest fluoride concentration. The groups GI and GII presented fluoride releases of 17.8 ppm and 30.0 ppm, respectively.

**Table 1:** Fluoride released in glass-ionomer cements

Periods days	Fluoride released				Recharged			
	GI mean (SD) ppm	GII mean (SD) ppm	GIII mean (SD) ppm	GIV mean (SD) ppm	GI mean (SD) ppm	GII mean (SD) ppm	GIII mean (SD) ppm	GIV mean (SD) ppm
> 1	17.8 (0.03)	30.0 (0.02)	32.6 (0.07)	17.4 (0.05)	–	–	–	–
> 2	10.0 (1.1)	3.2 (1.2)	22.0 (0.03)	5.9 (0.23)	–	–	–	–
> 6	7.7 (0.02)	2.5 (1.04)	3.7 (0.78)	2.3 (0.09)	–	–	–	–
10	5.6 (0.5)	2.2 (0.08)	3.2 (1.20)	1.5 (1.23)	–	–	–	–
31	2.4 (0.21)	1.9 (0.73)	2.2 (1.52)	1.2 (0.30)	–	–	–	–
32	–	–	–	–	77 (0.45)	28 (0.78)	41 (1.20)	33 (0.02)
90	23.3 (0.07)	11.9 (1.50)	15.0 (0.23)	10.2 (0.60)	–	–	–	–
180	30.9 (0.08)	18.8 (0.02)	24.4 (1.50)	21.5 (0.09)	–	–	–	–
181	–	–	–	–	81 (0.05)	51 (0.03)	41 (1.81)	36 (0.02)

SD, standard deviation; GI (Fuji IX extra); GII (Fuji IX); GIII (Ionofil Molar); GIV(Ketac Molar).

**Fig 1:**
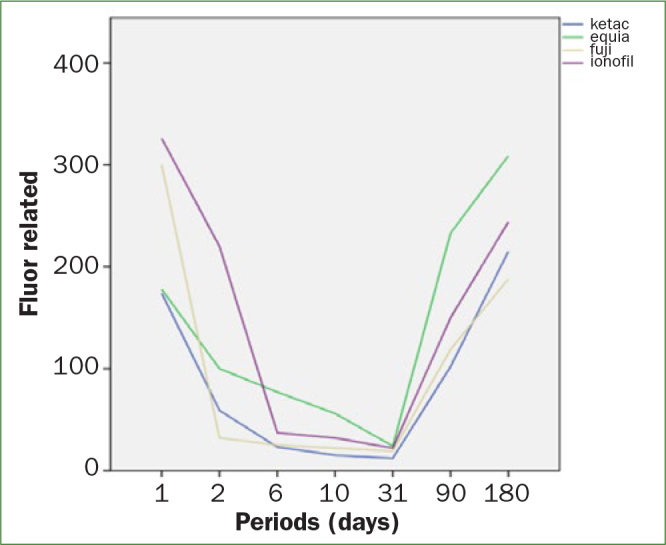
Fluoride released for each GICs evaluated versus time.

From the 2nd day, a slow, steady decline in fluoride release began and continued, with the exception of GII, which showed a marked decline to a value of 3.2 ppm. In Figure 1, the amount of fluoride released for each GICs evaluated versus time is clearly shown.

However, GI showed a lower but more constant release pattern, starting with 17.8 ppm and reaching up to 2.4 ppm until day 31. ANOVA test showed statistically significant differences between the amounts of fluoride of the four materials in the first 24 h (Table 2). However, the interaction between time and material shows that the fluoride release is not constant with time for all materials under study.

**Table 2:** Analysis of variance of fluoride released in the study groups

Source		Sum of squares	df	Mean-square	F	P
GI	Intergroup	599,333	7	85,619	256,857	0.004
(Fuji IX Extra)	Intragroup	0.667	2	0.333		
	Total	600,000	9			
GIII	Intergroup	3,805,833	7	543,690	12,264	0.077
(Ionofil Molar)	Intragroup	88,667	2	44,333		
	Total	3,894,500	9			
GIV	Intergroup	290,600	7	415,271	8475	0.110
(Ketac Molar)	Intragroup	98,000	2	49,000		
	Total	3,004,900	9			

ANOVA, analysis of variance.

**Fig 2:**
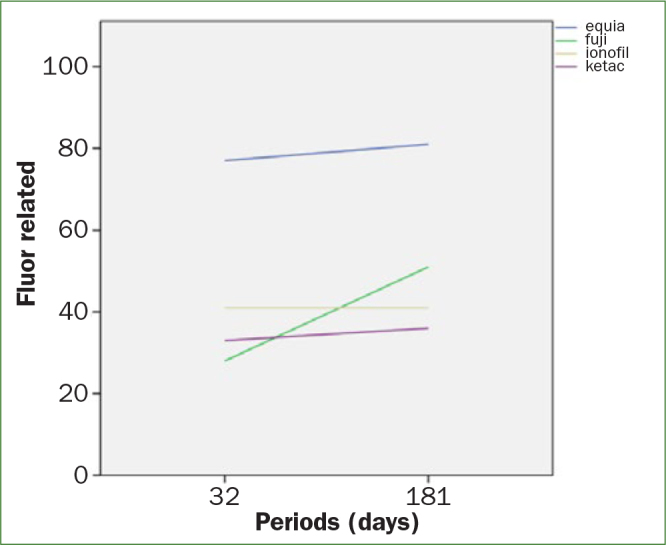
Fluoride release of each sample after being recharged.

On day 32, when recharges began with a fluorinated gel for 4 min, it can be seen that the recharge induced an increase in all GICs. In the same way, GI showed the highest fluoride release in day 32 when recharge started with a value of 77 ppm after the recharge. On day 90, GI has released again the highest amount of fluoride with a value of 23.3 ppm. In day 180, a second recharge was made, and the value for GI was 81 ppm. Figure 2 illustrates the fluoride release of each sample after being recharged. Therefore, GI presented an improved and sustained fluoride release during the study (Table 1).

Student t test was used to compare the fluoride release between the first and second recharge in each one of the study groups. Statistically significant differences were found when we compare the fluoride release in groups I (t = –16.95, p = 0.000) and IV (t = –2.644, p = 0.26) (Table 3).

**Table 3:** Student t test of fluoride released in the study groups

	Mean-square	Standard deviation	Confidence interval		t	gl	P
			Lower	Higer			
GI (Fuji IX Extra)	–23.000	4.295	–26.072	–19.928	–16.935	9	0.000
GII (Fuji IX)	–4.300	6.816	–9.176	0.576	–1.995	9	0.077
GIII (Ionofil Molar)	–4.300	6.816	–9.176	0.576	–1.995	9	0.077
GIV (Ketac Molar)	–3.300	3.917	–6.102	–498	–2.664	9	0.026

## DISCUSSION

According to some authors, the amount of fluoride release to prevent demineralisation and caries is not well documented.^21^ The values reported by different authors are between 0.02 and 0.2 ppm.

Based on our results, the GICs with conventional mixing were released between 32.6 and 17.4 ppm during the first 24 h, whereas the mechanical mixing GIC showed an average of 17.8 ppm in the same period. The higher fluoride release was observed in the first 24 h, these results match with those reported by Prabhakar et al^20^ where they found that in this period, the greatest fluoride release occurred. In this study, Fuji IX was evaluated and they reported values between 5.42 and 10.96 ppm, unlike in our results, where the value for the GII in conventional mixing was 30.0 ppm, and for mechanical mixing, it was 17.8 ppm.

Several authors mentioned that fluoride release commenced with an initial burst followed by a statistically significant decrease.^4,5,26^ Tiwari and Nandlal^24^ reported a marked decrease in conventional GICs in the mean fluoride released from day 1 to day 21.

However, Krämer et al^14^ report that after 14 days the GIC Ketac Molar showed a fluoride release with an average of 12 ± 8 ppm, contrary to what we found that Ketac Molar released just 1.5 ppm after 10 days of monitoring.

It can be observed that GIII with conventional mixing was the material that released the highest fluoride for the first 24 h, whereas GI with mechanical mixing was the material that presented a more constant fluoride release during the study.

## CONCLUSION

Some authors mention that the exposure to fluoride solutions cannot restore the initial fluoride release, and it is thought that the cause is the short time of recharge because the fluoride solution is in contact just with the superficial part of the sample. Ahn et al^2^ and Arbabzadeh et al^3^ carried out studies recharging with mouthwash for 20 min, but this method is clinically impractical because a patient cannot keep this topical fluoride agent during this time. In our study, all materials were recharged with sodium fluoride gel for 4 min, this period is established for this topical fluoride agent and is bearable for the patient, besides, we obtained positive results after recharging mainly in GI (mechanical mixing), where on day 32 a fluoride release of 77 ppm was shown and for the second recharge in day 181 (after 6 months), values of 81 ppm were shown. These results suggest that topical fluoride gel is a very important alternative for recharged fluoride-releasing materials, and the material of GI is an excellent option of treatment in patients that are at high risk in developing caries.
